# Supplementary material for: Preliminary Evidence for a Relationship between Elevated Plasma TNFα and Smaller Subcortical White Matter Volume in HCV Infection Irrespective of HIV or AUD Comorbidity
Source: Int J Mol Sci. 2021 May 7;22(9):4953. doi: 10.3390/ijms22094953 (PMC8124321; doi:10.3390/ijms22094953)
Supplement: Supplementary file 1 [file ijms-22-04953-s001.zip › ijms-1202208-supplementary.pdf]

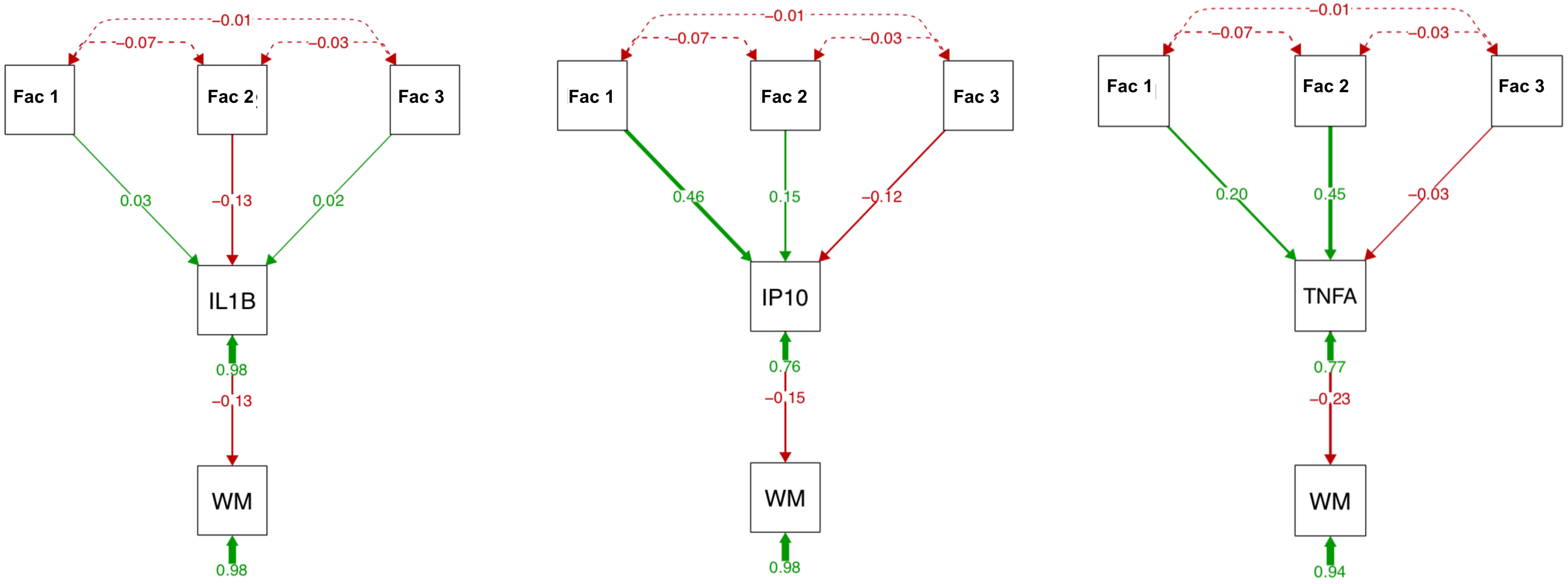

Supplementary Figure 1

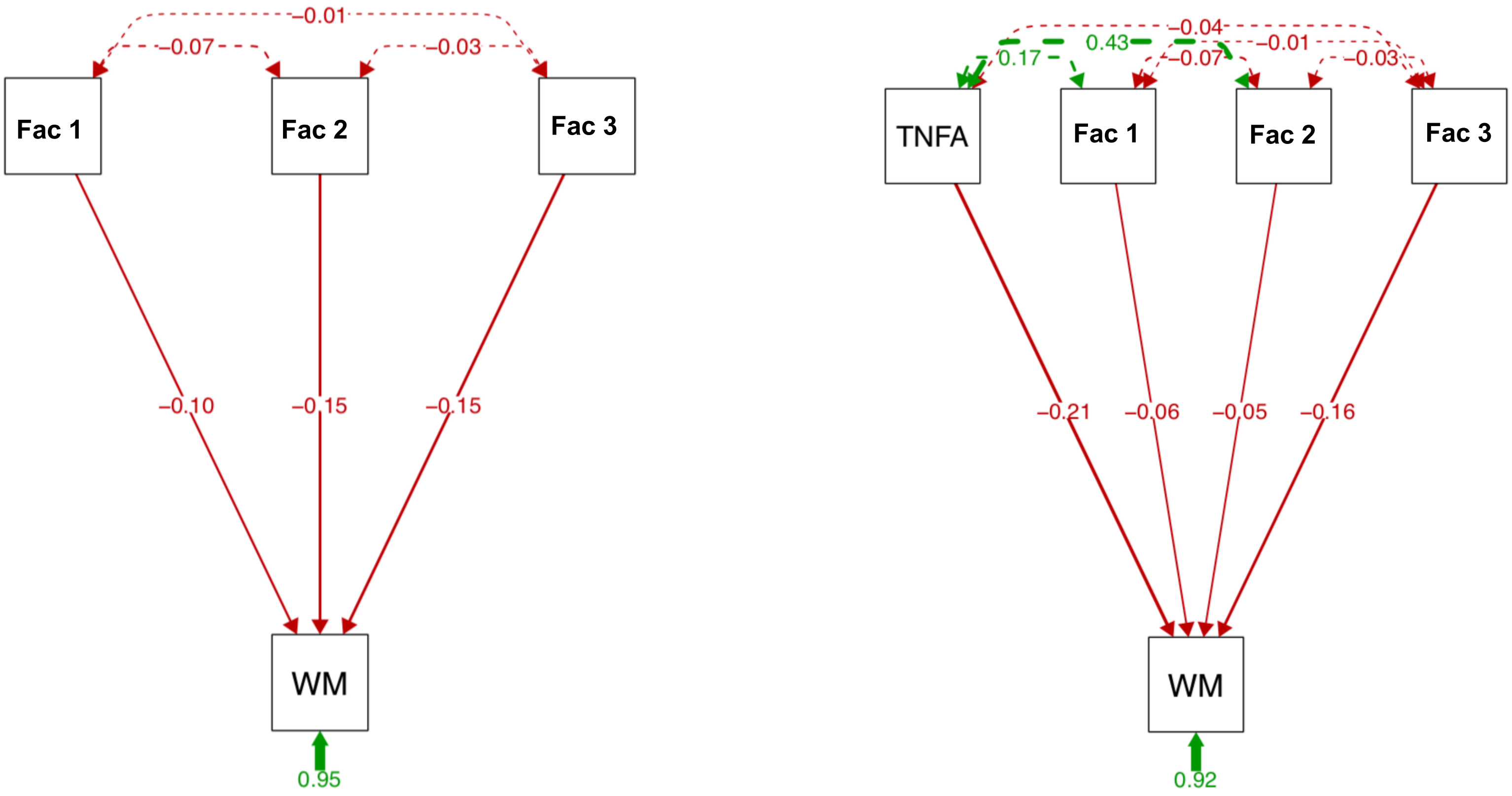

Supplementary Figure 2

Supplementary Table 1. Soluble protein levels by diagnostic pairs

Soluble Protein	*Con. vs. AUD*		*Con. vs. HIV*		*Con. vs. HIV+AUD*		*AUD vs. HIV*		*AUD vs. HIV+AUD*		*HIV vs. HIV+AUD*	
	t Ratio	p value	t Ratio	p value	t Ratio	p value	t Ratio	p value	t Ratio	p value	t Ratio	p value
CD40L	0.94	0.35	-0.37	0.71	-2.26	**0.03**	-0.94	0.35	-2.77	**0.006**	-0.99	0.33
EGF	-1.61	0.11	-0.69	0.49	-3.73	**0.0003**	0.20	0.84	-2.55	**0.01**	-1.64	0.11
EOTAXIN	0.17	0.86	2.42	**0.02**	0.75	0.46	2.36	**0.02**	0.64	0.53	-1.46	0.15
FGFB	-1.29	0.20	-1.83	0.07	-2.36	**0.02**	-0.84	0.40	-1.56	0.12	-0.78	0.44
FLT3L	-0.12	0.91	1.55	0.12	-0.27	0.79	1.75	0.08	-0.20	0.84	-1.65	0.10
Fractaline	0.28	0.78	-0.21	0.83	-1.84	0.07	-0.47	0.64	-2.34	**0.02**	-1.44	0.15
GCSF	-2.20	**0.03**	-2.59	**0.01**	-1.28	0.21	-0.99	0.33	0.23	0.82	0.85	0.40
GMCSF	-0.96	0.34	-2.48	**0.01**	-2.14	**0.03**	-1.95	0.05	-1.61	0.11	-0.04	0.97
GRO[‡]	2.03	**0.04**	0.65	0.52	0.16	0.88	-1.01	0.31	-1.52	0.13	-0.44	0.66
IFNA2	0.62	0.54	-0.64	0.52	-1.11	0.27	-1.23	0.22	-1.67	0.10	-0.48	0.63
IFNG[‡]	-1.46	0.15	-1.10	0.27	-2.11	**0.04**	0.07	0.94	-1.12	0.27	-0.96	0.34
IL1A	-2.64	**0.009**	-2.48	**0.01**	-3.48	**0.0007**	-0.15	0.88	-1.15	0.25	-0.83	0.41
IL1B	-1.94	0.05	-3.59	**0.0005**	-4.00	**0.0001**	-2.44	**0.02**	-3.01	**0.003**	-0.88	0.38
IL1RA	-0.27	0.79	0.44	0.66	-0.85	0.40	0.65	0.52	-0.67	0.50	-1.08	0.28
IL2	-1.38	0.17	-2.59	**0.01**	-2.75	**0.007**	-1.70	0.09	-1.95	0.05	-0.46	0.65
IL3	-0.70	0.49	-1.73	0.09	-2.20	**0.03**	-1.54	0.13	-2.20	**0.03**	-0.68	0.50
IL4	-0.99	0.33	-1.51	0.13	-3.75	**0.0003**	-0.76	0.45	-3.30	**0.001**	-2.11	**0.04**
IL5	-1.68	0.10	-0.58	0.56	-1.97	0.05	0.63	0.53	-0.74	0.46	-1.03	0.30
IL6	-0.92	0.36	-0.46	0.65	-2.43	**0.02**	0.30	0.77	-1.92	0.06	-1.75	0.08
IL7	-1.08	0.28	-1.97	0.05	-1.59	0.11	-1.31	0.19	-0.90	0.37	0.07	0.94
IL8	-0.51	0.61	1.26	0.21	0.79	0.43	1.83	0.07	1.30	0.20	-0.40	0.69
IL9	-1.49	0.14	-2.48	**0.01**	-3.43	**0.0008**	-1.45	0.15	-2.59	**0.01**	-0.77	0.44
IL10	-0.87	0.39	-1.14	0.26	-1.70	0.09	-0.52	0.61	-1.20	0.23	-0.48	0.64
IL12P40[‡]	-1.60	0.11	-2.90	**0.004**	-1.65	0.10	-1.79	0.08	-0.44	0.66	0.84	0.41
IL12P70[‡]	-0.05	0.96	-1.64	0.10	-1.12	0.26	-1.69	0.09	-1.12	0.26	0.22	0.83
IL13	-1.80	0.07	-2.04	**0.04**	-3.38	**0.0009**	-0.68	0.50	-2.15	**0.03**	-0.93	0.35
IL15	-0.88	0.38	-2.38	**0.02**	-1.68	0.10	-2.10	**0.04**	-1.18	0.24	0.30	0.76
IL17	-1.94	0.05	-1.12	0.26	-3.24	**0.002**	0.49	0.63	-1.97	0.05	-2.00	**0.05**
IP10‡	0.02	0.99	3.85	**0.0002**	4.81	**<.0001**	3.94	**0.0002**	4.90	**<.0001**	1.29	0.20
MCP1[‡]	0.57	0.57	1.68	0.10	0.50	0.62	1.29	0.20	0.06	0.95	-1.00	0.32
MCP3	-2.03	**0.04**	-2.12	**0.04**	-2.11	**0.04**	-0.53	0.60	-0.58	0.56	-0.06	0.95
MDC[‡]	1.18	0.24	0.37	0.71	0.86	0.39	-0.48	0.63	-0.09	0.93	0.35	0.73
MIP1A	-1.30	0.20	-0.16	0.88	1.02	0.31	1.13	0.26	1.72	0.09	1.11	0.27
MIP1B	-1.05	0.29	-0.40	0.69	0.33	0.74	0.59	0.56	1.02	0.31	0.60	0.55
PDGFAA	1.41	0.16	0.91	0.36	0.41	0.68	-0.25	0.80	-0.89	0.38	-0.51	0.61
PDGFBB	2.71	**0.007**	1.08	0.28	0.84	0.40	-1.24	0.22	-1.52	0.13	-0.23	0.82
RANTES[‡]	1.53	0.13	2.13	**0.04**	1.38	0.17	0.67	0.51	-0.13	0.90	-0.78	0.44
TGFA	-0.15	0.88	0.46	0.65	-0.19	0.85	0.58	0.56	-0.09	0.93	-0.55	0.58
TNFA[‡]	1.93	0.06	5.61	**<.0001**	3.80	**0.0003**	4.48	**<.0001**	2.60	**0.01**	-1.39	0.17
TNFB	-1.00	0.32	-1.05	0.30	-2.42	**0.02**	-0.38	0.71	-1.82	0.07	-0.93	0.35
VEGF	-1.15	0.25	-1.00	0.32	-2.08	**0.04**	-0.24	0.81	-1.39	0.17	-0.83	0.41

Supplementary Table 2. Soluble protein levels by HCV status

Soluble Protein	*HCV+ vs HCV-* t Ratio	p value
**CD40L**	-0.05	0.96
**EGF**	-1.53	0.13
**EOTAXIN**	-0.11	0.91
**FGFB**	-2.12	**0.04**
**FLT3L**	0.33	0.74
**Fractaline**	-1.32	0.19
**GCSF**	-0.40	0.69
**GMCSF**	0.05	0.96
**GRO‡**	-1.86	0.07
**IFNA2**	0.90	0.37
**IFNG‡**	-1.14	0.26
**IL1A**	-0.87	0.39
**IL1B**	-2.38	**0.02**
**IL1RA**	0.88	0.38
**IL2**	-0.86	0.39
**IL3**	-0.83	0.41
**IL4**	-0.56	0.57
**IL5**	-1.62	0.11
**IL6**	-0.99	0.33
**IL7**	-0.47	0.64
**IL8**	2.48	**0.02**
**IL9**	-0.65	0.52
**IL10**	0.40	0.69
**IL12P40‡**	0.18	0.86
**IL12P70‡**	-1.46	0.15
**IL13**	-1.02	0.31
**IL15**	-0.80	0.42
**IL17**	-1.43	0.16
**IP10‡**	7.22	**<.0001**
**MCP1‡**	-0.12	0.91
**MCP3**	-0.19	0.85
**MDC‡**	0.32	0.75
**MIP1A**	-0.93	0.36
**MIP1B**	0.06	0.95
**PDGFAA**	-0.31	0.75
**PDGFBB**	-0.13	0.90
**RANTES‡**	1.21	0.23
**TGFA**	0.04	0.97
**TNFA‡**	4.19	**<.0001**
**TNFB**	0.11	0.91
**VEGF**	-1.40	0.17
